# Knee Joint Rescue in a Traumatic Amputation With Coverage by a Fillet-Free Flap From the Amputated Leg

**DOI:** 10.7759/cureus.42917

**Published:** 2023-08-03

**Authors:** Nicolas Cavadore, Ugo Lancien, Vincent Crenn, Julien Verdier, Pierre Perrot

**Affiliations:** 1 Plastic and Reconstructive Surgery, University Hospital of Nantes, Nantes, FRA; 2 Orthopaedics and Traumatology, University Hospital of Nantes, Nantes, FRA

**Keywords:** below-the-knee amputation, microsurgery, posterior tibial pedicle, traumatic amputation, fillet free flap

## Abstract

Traumatic amputation is a severe injury that requires urgent surgical care. A fillet-free flap from the amputated limb is the most conservative way to ensure proper coverage of the stump when replantation is not possible. We report the case of a male patient who suffered from a traumatic limb amputation in a motorcycle accident. A free fillet flap from the posterior compartment of the leg carrying the posterior tibial pedicle, the soleus muscle, and skin tissue harvested from the amputated limb was performed to cover the amputation stump and thus allow preservation of the knee joint. In our case report, the patient conserved almost maximal knee joint range of motion (130°). He regained the ability to walk only two months after the initial trauma. Prosthetic fitting occurred quickly and without any particular issues throughout the process. Regarding quality of life, after one year, our patient had a five-level EQ-5D version (EQ-5D-5L) score of 21,221, and his 36-Item Short Form Survey (SF-36) score was divided between the five components in 85 points in physical functioning, 100 points in role limitations due to physical health, 100 points in role limitations due to emotional problems, 50 points in energy/fatigue, 68 points in emotional well-being, 75 points in social functioning, 45 points in pain, and 95 points in general health. This technique helped provide good coverage of the stump while preserving a functional knee joint, allowing for effective prosthetic fitting in the future and therefore optimizing our patient’s quality of life.

## Introduction

Below-the-knee amputations can occur in severe trauma situations, and proper coverage of the stump is crucial to ensure healing, prosthesis fitting, and patient comfort. They represent 10-20% of all amputations [1]. Several coverage options are available, including a skin graft, local, and free flaps or, in our case, the use of a fillet-free flap [2]. The level of lower limb amputation remains a major functional issue for the patient, and preserving the knee joint is essential for gait rehabilitation and prosthetic fitting [3]. In cases of traumatic limb amputation, it is often difficult to perform only a below-the-knee amputation due to extensive soft tissue loss, which prevents optimal stump coverage. However, an above-the-knee amputation involves a significant increase in morbidity and mortality among patients [4].

We report a case of below-the-knee amputation in a traumatic context with stump coverage by a soleus muscle fillet-free flap from the amputated leg to preserve the knee joint.

## Case presentation

A 34-year-old patient was involved in a high-kinetic motorcycle accident in March 2022, with a 25-m projection from his vehicle, his right leg becoming severed. Initial whole-body CT scan showed a severely damaged and open wound at the knee-level stump of the right lower limb, with joint communication and popliteal vascular amputation, but no active hemorrhagic bleeding. The other injuries were multiple spine thoracic fractures from T4 to T7 (with only the T4 fracture being unstable) and some bilateral pulmonary contusions in the inferior lobes.

**Figure 1:**
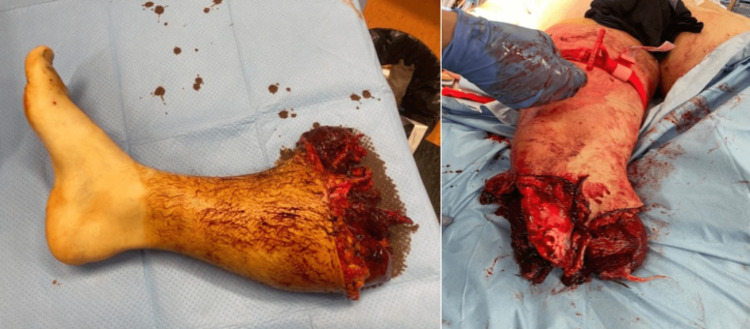
Amputated leg and traumatic amputation stump.

Local lesion assessment revealed a complete amputation of the leg at its proximal third, with the impossibility of replantation because of vascular and nervous stripping. The skin tissues were dissected at the joint space, without obvious joint violation, except for a 1-cm exposure of the infrapatellar fat pad. There was also an avulsion of the head of the fibula (Figure 1).

An immediate below-the-knee amputation closure was not possible. After surgical exploration and due to the quality and viability of the soft tissue and vascular pedicles in the amputated part but also the patient's age, the delay in performing the procedure (four hours), and the functional benefit of a below-the-knee versus an above-the-knee amputation, we decided to perform a fillet free flap from the amputated leg, which would allow for coverage of the resulting stump.

The surgery was carried out in several stages. Firstly, we dissected the amputated leg and located the relatively healthy tibiofibular trunk on the amputated limb. Both gastrocnemius muscles were lost in the trauma, but the soleus muscle appeared viable. Therefore, we decided to harvest the soleus muscle-cutaneous flap on the posterior tibial vessels. Initially, we approached the medial side of the leg and located the vascular pedicle in a healthy retro-malleolar internal area. We preserved the maximum amount of skin envelope to facilitate the coverage of the amputation stump. The muscle compartments were opened, and the soleus muscle was isolated and harvested on its vascular pedicle. 

**Figure 2:**
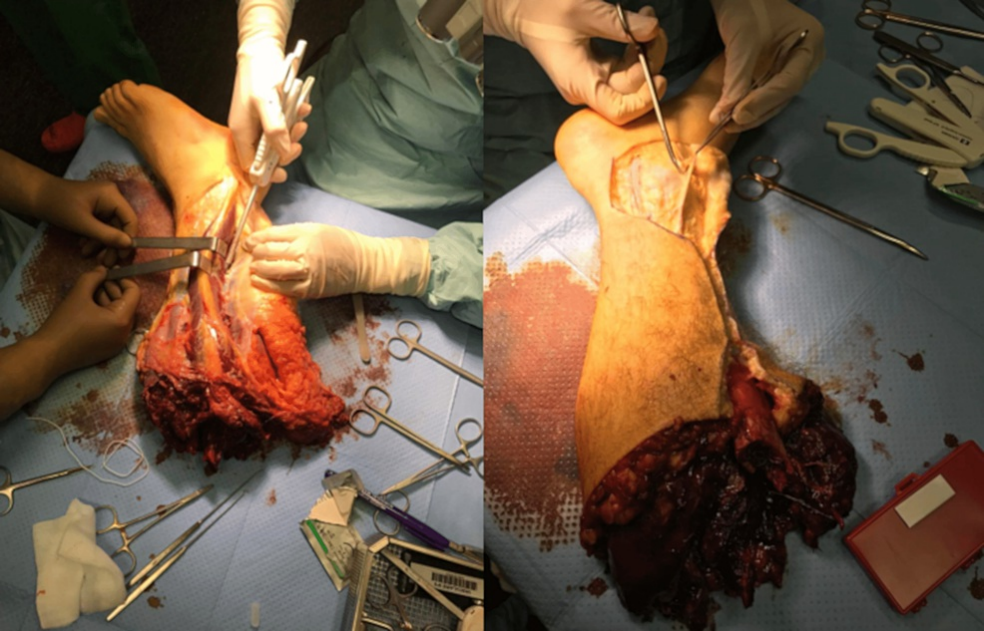
Dissection of the posterior tibial pedicle and the great saphenous vein.

The pedicle was then raised from distal to proximal, with all arteries not supplying the flap being ligated. We also harvested the maximum amount of skin and cut the Achilles tendon. The great saphenous vein was carefully dissected and put on hold on clips as well (Figure 2). Then, we debrided the skin and muscle before performing a cut with ligation of the common fibular and tibial nerves. Thereafter, we cut the tibia at 10 cm from the joint space (with a cut angle of 35°). A myodesis procedure was performed using a drill. We proceed with the excision of the head of the fibula and reinsertion of the external plan onto an Arthrex® anchor.

Finally, we began the microvascular anastomose stage by performing an end-to-end anastomosis of the tibiofibular trunk on the lower popliteal artery and an end-to-end anastomosis of the popliteal vein with the proximal posterior tibial vein. Good vascular patency was immediately observed after releasing the limb tourniquet.

**Figure 3:**
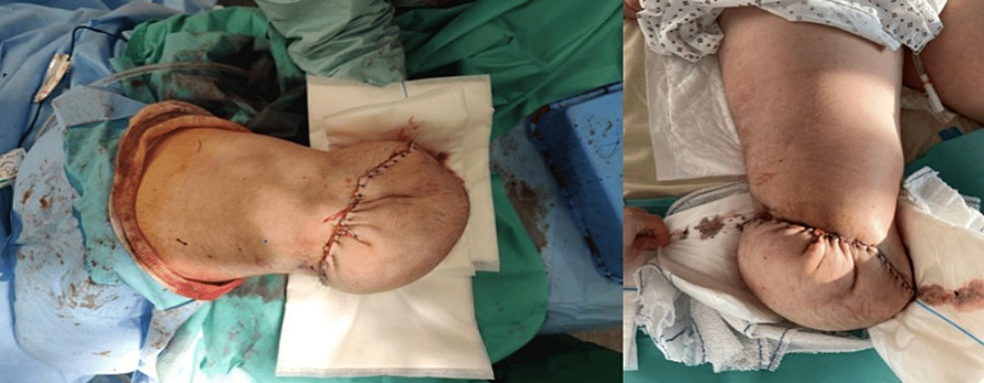
Amputation stump after suture of the skin sheath.

At last, we sutured the soleus muscle to both gastrocnemius muscles before completing skin closure (Figure 3).

At one month, the volume of the stump had significantly decreased, and the range of motion of the knee joint was almost maximal (130°).

Two months after the accident, the scar tissue trophicity was excellent, and the first training prosthesis allowed for walking between parallel bars with an initial sensation of instability in the right knee, probably due to sensory disturbances. There was distal anesthesia in the flap area, which was not interfering with the prosthesis fitting.

Just over three months after the trauma (during his hospitalization in the rehabilitation center), the patient walked without any assistive device for several hundred meters, on all types of terrain (inclines and stairs) with a six-minute walk test (6MWT) of 474 m. His driving test was already favorable. In hydrotherapy, he started swimming breaststroke and crawl stroke again without any major difficulty. In adapted physical activity, he covered 2 km without difficulty in Nordic walking.

**Figure 4:**
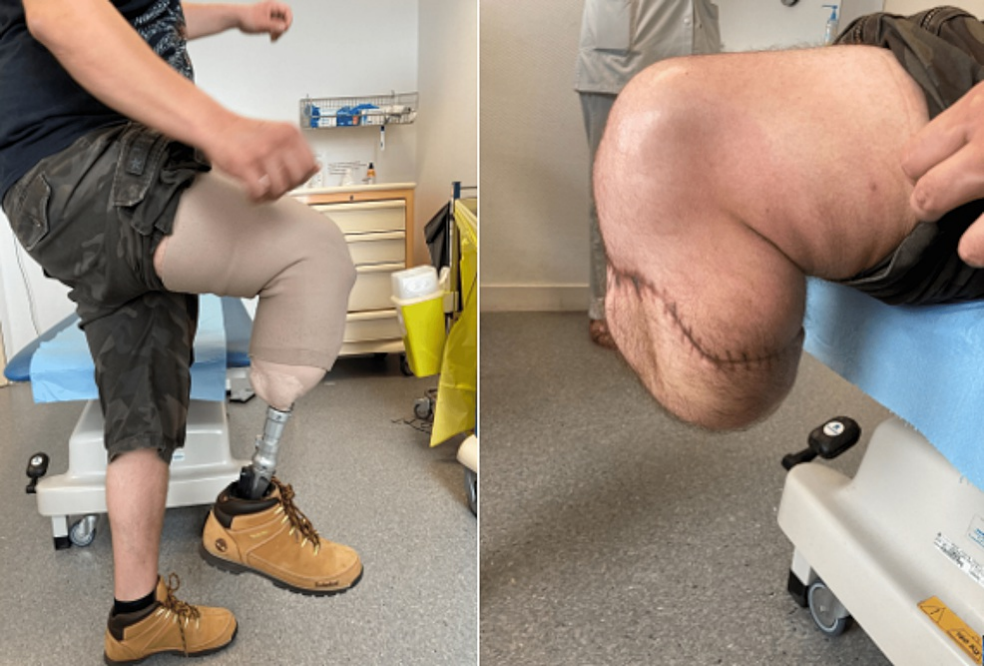
Stump with prosthesis fitted and without, with an almost maximal range of motion (130°).

Five months after the trauma (one month after discharge from rehabilitation), the patient used his prosthesis daily without any assistive device, over an unrestricted perimeter. Regarding the scar, the amputation stump was completely healed, and its volume had significantly decreased, with no conflict with the prosthesis (Figure 4).

One year after the trauma, the flap trophicity was excellent, and the scars were minimally inflamed and perfectly flexible. The patient walked daily with his prosthesis without any assistive device over an unrestricted perimeter, without limping or pain. There was no visible skin conflict or suffering during the prosthesis fitting. However, distal anesthesia of the stump persisted. He was completely self-sufficient at home and was about to return to work in a month. We also evaluated the quality of life and mental health of our patients.

His EQ-5D-5L had a score of 21,221 (2 points in mobility, 1 point in self-care, 2 points in usual activities 2 points in pain/discomfort, and 1 point in anxiety/depression).

Regarding his SF-36, he had 85 points in physical functioning, 100 points in role limitations due to physical health, 100 points in role limitations due to emotional problems, 50 points in energy/fatigue, 68 points in emotional well-being, 75 points in social functioning, 45 points in pain, and 95 points in general health.

## Discussion

The concept of fillet flaps is reported in the surgical literature since the 1990s, involving their utilization either as pedicle or free flaps. Classifications have been described by several authors, which facilitate surgical planning [5].

The use of a fillet-free flap from the amputated leg to cover a below-the-knee amputation stump is a surgical technique that can be used in cases of severe traumatic injuries requiring effective coverage, as well as rapid and optimal prosthesis fitting [6]. This is an emergency surgery that requires trained medical and paramedical teams [7].

In the oncologic context, fillet flap coverage has also been described, particularly in thoracic reconstruction after tissue loss requiring shoulder disarticulation [8].

This technique has the advantage of allowing rapid healing and good functionality of the affected leg [9]. Still, there are some limitations, such as the long operating time, needed for the surgery and the risk of free flap failure. In our case report, the time before the first ambulation (i.e. two months) is far shorter than the mean duration of the first ambulation with a prosthesis [10].

Regarding our patient, the use of the spared limb was deemed appropriate due to the good preservation of the musculocutaneous tissue, which allowed for a conservative below-the-knee amputation. Typically, patients with below-the-knee or through-the-knee amputations have a greater ability to walk 500 m than those with above-the-knee (p=0.0035) [11]. The procedure was successful, and healing occurred without complications. The patient quickly regained satisfactory functionality of the affected leg by maintaining almost maximal knee joint amplitude (130°) [11]. Concerning the quality of life, our patient had a higher score at his EQ-5D-5L [2]. His 36-Item Short Form Survey (SF36) score was mainly superior, except for the pain [12]. However, the study population was way older than our patient (average age 65.36 ± 13.64) and was suffering mostly from vascular disease [12]. Those variables might affect the way pain is perceived. We selected those tests in our case report because they are standardized forms that are widely used in the literature to assess the quality of life and mental health in patients with amputated limbs [2-12].

It is also important to underline that the feasibility of this type of surgery depends on the initial damage caused by the trauma. The main limitation in the outcomes of this type of flap is the distal anesthesia of the stump, which can result in difficulties during prosthesis fitting, particularly by masking a cutaneous conflict between the flap and the prosthesis (which can lead to the formation of a pressure sore, for example) [13]. We did not report any wound healing disorder or pressure sore of any type since he started using his prosthesis. Thus, this requires careful monitoring of patients who have undergone such a procedure to detect any signs of possible complications [10].

One possibility to avoid that outcome could have been a nerve anastomosis between a sensitive nerve in this area (e.g. the saphenous or the sural nerve) and the tibial nerve’s proximal stump, to regain flap sensitivity [7]. In our case, it was impossible due to the initial nervous stripping. Still, the result of this type of surgery is variable and depends on many other factors such as the patient’s age, vascular restitution, degree of nerve contusion, and the number of sectioned tendons [14].

## Conclusions

In conclusion, the use of a fillet-free flap is a valuable surgical technique for providing effective coverage and rapid healing of severe traumatic lower limb injuries requiring amputation. Although this technique offers good functionality of the affected limb by allowing for a conservative below-the-knee amputation, its feasibility depends on the initial extent of the trauma and microsurgical team availability. One main potential limitation of this type of surgery is distal anesthesia of the stump, which requires careful patient monitoring to detect any signs of complications when adjusting the prosthesis. Despite these limitations, the success of this operation and its outcomes underlines the potential benefits of using a fillet-free flap of a spared limb in specific cases of traumatic amputation.
